# Identifying Key Factors for Predicting the Age at Peak Height Velocity in Preadolescent Team Sports Athletes Using Explainable Machine Learning

**DOI:** 10.3390/sports12110287

**Published:** 2024-10-22

**Authors:** Nikolaos-Orestis Retzepis, Alexandra Avloniti, Christos Kokkotis, Maria Protopapa, Theodoros Stampoulis, Anastasia Gkachtsou, Dimitris Pantazis, Dimitris Balampanos, Ilias Smilios, Athanasios Chatzinikolaou

**Affiliations:** School of Physical Education and Sport Science, Democritus University of Thrace, 69100 Komotini, Greece; nretzepi@phyed.duth.gr (N.-O.R.); ckokkoti@affil.duth.gr (C.K.); mprotopa@phyed.duth.gr (M.P.); tstampou@phyed.duth.gr (T.S.); anasgkac1@phyed.duth.gr (A.G.); dpantazi@phyed.duth.gr (D.P.); ismilios@phyed.duth.gr (I.S.); achatzin@phyed.duth.gr (A.C.)

**Keywords:** children, peak height velocity, classification, feature selection, interpretation

## Abstract

Maturation is a key factor in sports participation and often determines the young athletes’ characterization as a talent. However, there is no evidence of practical models for understanding the factors that discriminate children according to maturity. Hence, this study aims to deepen the understanding of the factors that affect maturity in 11-year-old Team Sports Athletes by utilizing explainable artificial intelligence (XAI) models. We utilized three established machine learning (ML) classifiers and applied the Sequential Forward Feature Selection (SFFS) algorithm to each. In this binary classification task, the logistic regression (LR) classifier achieved a top accuracy of 96.67% using the seven most informative factors (Sitting Height, Father’s Height, Body Fat, Weight, Height, Left and Right-Hand Grip Strength). The SHapley Additive exPlanations (SHAP) model was instrumental in identifying the contribution of each factor, offering key insights into variable importance. Independent sample *t*-tests on these selected factors confirmed their significance in distinguishing between the two classes. By providing detailed and personalized insights into child development, this integration has the potential to enhance the effectiveness of maturation prediction significantly. These advancements could lead to a transformative approach in young athletes’ pediatric growth analysis, fostering better sports performance and developmental outcomes for children.

## 1. Introduction

Maturation, the process of becoming an adult, involves significant changes in body size, composition, and physical attributes, especially during childhood and puberty [1]. Youth athletes are categorized into annual age groups to minimize possible developmental differences and create fair competition conditions. However, in that way, there is a greater possibility for athletes of higher biological age to be considered talented since they gain a temporary performance advantage [2]. It is well documented that as players mature, they improve their performance, and for athletes that train and compete in the same chronological category, the potential mismatch will be increased [3,4]. These findings highlight the importance of reconsidering and establishing more specific and accurate factors that identify talent selection.

One crucial aspect of maturation is Peak Height Velocity (PHV), which refers to the period of maximum growth rate in stature. During PHV, girls typically experience height increases of about 8–9 cm per year, while boys see increases of about 10–11 cm per year [5]. This peak growth period occurs at different chronological ages for boys and girls—between 9 and 13 years for girls and 12–16 years for boys [6]. Notably, a few months before and during PHV, there is a rapid elongation of the arms and legs compared to the trunk [7], along with increases in body weight [8] and muscle mass. Furthermore, it is important to account for the fact that body segments grow at different rates and times. For example, the lower limbs experience accelerated growth earlier in life compared to the trunk. Specifically, the growth spurt for lower body dimensions occurs before PHV, while for the upper body, it happens after the PHV period [9]. Considering these variations, sitting height and leg length have been established as two key indicators of maturation, individually and in proportion [9,10]. Additionally, body mass increases significantly shortly after the growth spurt in height, presenting another benchmark of maturation. However, body mass is often considered in terms of body composition (fat-free mass and fat mass). An adolescent spurt tends to occur in fat-free mass, while fat mass increases more gradually [11]. Adolescents post-PHV exhibit greater muscle power, speed, and aerobic capacity compared to those in the pre-PHV and circa-PHV stages [5]. However, negative effects may also occur alongside the positive outcomes of the adolescent growth spurt. This phenomenon, referred to as ‘adolescent awkwardness’, describes a temporary decrease in motor control and, ultimately, in performance, due to the accelerated growth of limbs [12]. During puberty, this condition may increase the risk of injury, emphasizing the importance of timely assessment of maturity status [13].

In addition to physiological factors, other elements such as nutrition, genetics, hormones, and environmental cues can influence the maturation process and trigger various physical and functional changes in the body [14,15]. Along with these factors, physical activity plays a critical role in young athletes’ growth, development, and health, as it enhances morphology, physiology, and functional parameters [16]. Throughout childhood and adolescence, the extent of these changes is directly influenced by the interaction between maturation and training exposure. As a result, an athlete’s response to training load will vary according to the development of body systems, including sexual characteristics [17].

Assessing the tempo and timing of maturity, rather than relying solely on chronological age, is essential for determining an individual’s maturity status [18]. Due to the significant variability in maturity among children and adolescents of the same chronological age, more precise methods are needed to classify individuals as early or late maturers [1,19]. Several well-established methods are used to assess child growth, including skeletal age, stage of puberty, testicular volume, age at menarche [6], dental age [18], and predicted adult height [20].

One of the most practical and reliable methods is the predicted maturity offset, which estimates the time before or after PHV and the age at PHV. These models are based on sex-specific equations that require measurements of chronological age, height, weight, sitting height, and leg length [21]. Mixed-longitudinal studies have provided deeper insights into growth milestones, constructing height velocity charts and identifying critical growth phases such as the onset of the adolescent growth spurt and PHV. This detailed analysis aids in understanding growth dynamics and guiding interventions to support healthy development in children [22].

In addition to the aforementioned points, recent advancements in artificial intelligence (AI) have significantly impacted the prediction and analysis of maturation. AI techniques are increasingly being used to predict adult height and assess growth patterns based on early childhood data. For instance, Shmoish et al. [23] utilized machine learning (ML) models, including random forest regressors, to predict adult height from early height and weight data. Their approach achieved high prediction accuracy by incorporating features such as sex and early childhood growth measurements. Additionally, a study by Zhang et al. [24] highlighted the clinical application of AI in longitudinal image analysis of bone age among Growth Hormone Deficiency (GHD) patients, showcasing how AI can aid in more precise and individualized growth predictions. These advancements highlight the potential of AI in revolutionizing pediatric growth analysis, providing precise and individualized predictions that can enhance both health outcomes and maturation.

Despite the significance of age at PHV and its implications for adolescent growth, there is a notable gap in understanding the specific factors that differentiate between children who fall below and above the median predicted age at PHV by using AI. Traditional predictive models often lack the precision to identify these key differentiators due to the high variability among individuals. While limited studies have employed AI for growth predictions, the focus on explainable AI techniques to distinguish between these two groups is unexplored. The present study employs explainable AI (XAI) to identify key factors that differentiate children with predicted age at PHV below the median from those above the median. One of the main shortcomings of conventional methods is that they often operate as ‘black boxes’, offering limited insights into the relative importance of growth parameters. This creates a need for more interpretable models that can guide targeted interventions. XAI adds new value by providing clear, interpretable predictions, allowing for a deeper understanding of how various growth factors contribute to maturation. Unlike traditional models, XAI offers transparency, making it possible to identify and differentiate key predictors with greater accuracy and clarity [25,26,27,28]. This methodology allows for more accurate, interpretable, and actionable insights into the growth dynamics during the pre-adolescent period, facilitating better-targeted interventions.

Hence, our study aims to confirm whether XAI can significantly enhance predictions of age at PHV in pre-adolescent athletes by identifying the most important growth factors. Specifically, we aim to answer the following research questions: (i) What are the most significant predictors that differentiate children with predicted age at PHV ≤ median from those with PHV > median? (ii) How can explainable machine learning models improve our understanding of these predictors? (iii) How can this information support pediatricians, coaches, and parents in monitoring and intervening in athlete development? These advancements underscore the potential of XAI to revolutionize pediatric growth analysis, providing precise and individualized predictions that can enhance both health outcomes and sports performance.

## 2. Materials and Methods

### 2.1. Study Design

All measurements were conducted at the Laboratory of Physical Education and Sport within the School of Physical Education and Sport Science at Democritus University of Thrace. Each participant was assessed during a single visit, ensuring consistency by performing all evaluations at the same time of day. The assessments included anthropometric measurements, body composition, and upper and lower limb strength and power evaluations. The anthropometric data collected comprised standing and sitting heights, estimated leg length, and body weight. These measurements were used to calculate Peak Height Velocity (PHV) using the equation developed by Mirwald et al. [18]. Specifically, by calculating the median of the Mirwald score for our sample, we divided it into children with predicted age at PHV ≤ median and children with predicted age at PHV > median.

Additional anthropometric parameters included body mass index (BMI) and body composition metrics such as body fat percentage, body fat mass (kg), and lean mass (kg). To evaluate physical performance, two specific tests were employed, isometric handgrip strength and countermovement jump height. These tests were chosen for their relevance in assessing upper and lower limb strength, respectively. All measurements and tests were administered by experienced personnel to ensure accuracy and reliability. Prior to participation, parents or legal guardians received comprehensive information about the study, both verbally and in writing. Written informed consent was obtained from each participant’s parent or guardian. The study received ethical approval from the Research Ethics Committee of Democritus University of Thrace, under reference number DUTH/EHDE/36699/257-25 February 2022.

### 2.2. Participants

The study sample consisted of 93 male youth athletes engaged in soccer, basketball, and volleyball. Each participant had a minimum of two years of training experience, practiced at least three times per week, and had remained injury-free for the preceding six months. These athletes, who were 6th-grade students during the 2023–2024 season, also competed in U12 and U13 teams in regional championships. Table 1, Table 2 and Table 3 present the subjects’ characteristics.

### 2.3. Procedures

#### 2.3.1. Anthropometric Measurements

Participants’ and their parents’ standing heights, as well as sitting heights for participants, were measured by experienced researchers in the field of pediatrics and exercise to the nearest mm two times, and average values were recorded [29]. Lower limb length was derived by subtracting sitting height from standing height. Weight was measured using a Seca weight scale, with participants barefoot, to the nearest 0.1 kg [4]. Anthropometric measurements were performed according to Mirwald et al. [18]. Body Mass Index (BMI) was calculated using the formula BMI = weight (kg)/height^2^ (m). Body composition, including body fat mass and lean mass, was assessed using a Charder MA801 body composition analyzer from Taiwan. Chronological age was determined by subtracting the date of birth from the measurement date.

#### 2.3.2. Upper Limb Strength Assessment

Isometric upper limb strength was assessed with a Charder MG 4800 medical handgrip dynamometer. The test was performed while the participant was seated, with the shoulder of the tested arm fully adducted and touching the side of the trunk, the elbow flexed at 90 degrees, and the forearm and wrist in a neutral position [30]. Each participant completed a warm-up trial followed by two maximal effort trials, with the best result recorded.

#### 2.3.3. Lower Limb Power Assessment

Lower limb power was evaluated using countermovement jumps (CMJ) both without and with arm swing (CMJas) on a Swift Performance EZE JUMP contact time jumping mat. Before testing, participants underwent a standardized warm-up, including five minutes of low-intensity running and dynamic stretching, followed by three sub-maximal jumps. For the CMJ without arm swing, participants placed their hands on their hips, performed a squat, and then executed a maximal jump [31]. For the CMJas, participants began with elbows flexed at 90 degrees, positioned in front of the body, and moved their arms at maximum speed during the jump [32]. The best result from two maximal trials was recorded.

### 2.4. Outcome Variable

The dependent variable, Age at Peak Height Velocity (PHV), was calculated using equations from Mirwald et al. [18], which predict the time (in years) that an athlete is from reaching their PHV.

Maturity offset = −9.236 + (0.0002708 × leg length × sitting height) − (0.001663 × age × leg length) + (0.007216 × age × sitting height) + (0.02292 × (weight/height) × 100)

By employing these standardized measurements and assessments (14 predictors), we aim to accurately identify the key factors that differentiate between groups with PHV below and above the median, enhancing our understanding of growth patterns at age 11.

### 2.5. Machine Learning Workflow

Power Analysis. A power analysis indicated that a total sample size of 88 participants (44 per group) would be necessary to achieve at least 80% sensitivity and specificity, with a margin of error of 0.10 [33]. Given that we have at least 46 participants in each group, our sample size meets this requirement, ensuring sufficient statistical power for the analysis.

Data Preprocessing. In this study, the issue of missing data, confined to continuous variables and constituting no more than 5.5% of the dataset, was addressed by using the mean strategy. To ensure consistency across all phases, the normalization of data was carried out using StandardScaler library, which standardizes features by eliminating the meaning and scaling them to have unit variance. This preprocessing step was crucial during both the feature selection (FS) phase and the ML model training phase.

Feature Selection. For FS, we utilized Sequential Forward Feature Selection (SFFS) combined with 10-fold Stratified Cross-Validation. SFFS is a powerful ML technique that iteratively builds a feature subset by adding one feature at a time, with each iteration selecting the feature that optimally enhances the model’s performance. The use of stratified cross-validation ensures balanced class distributions, which is particularly important for imbalanced datasets. The SFFS approach offers several advantages, as follows: it improves predictive accuracy while maintaining robustness, identifies the most relevant variables step-by-step, potentially reducing model complexity and overfitting, and tailors the selected feature subset to the specific problem at hand, enhancing model generalization and interpretability.

ML Learning Process. In our binary classification task, we employed three advanced ML algorithms, Random Forest (RF), Logistic Regression (LR), and Neural Networks (NNs). This multi-algorithmic approach was chosen for several reasons. It allows us to assess the robustness of our models by comparing results across different algorithms, mitigates algorithmic biases inherent to any single method, and provides insights into the sensitivity of the task to different features. Given the absence of a gold standard for selecting the best ML algorithm for this task, employing multiple algorithms enhances the credibility and generalizability of our findings. The FS process was conducted separately for each classifier to account for their unique interactions with the features’ predictive power. To ensure robust model performance and minimize overfitting, we implemented a 10-fold stratified cross-validation strategy. Furthermore, hyperparameter tuning was performed through 10-fold stratified cross-validation within the training set to fine-tune the models for optimal configurations. This thorough approach enabled us to extract meaningful insights and achieve high predictive accuracy in our complex task, demonstrating the versatility and adaptability of these ML techniques.

Performance Metrics. To rigorously assess the testing performance of our ML models, we employed a comprehensive suite of metrics such as the accuracy, which represents the proportion of true results (both true positives and true negatives) among the total cases examined; the precision, which is calculated as the proportion of true positive identifications out of all positive identifications made by the model, reflecting the reliability of positive predictions; the recall (Sensitivity), measuring the model’s capability to identify all relevant instances, indicated by the proportion of actual positives correctly identified; the F1-score, which provides a harmonic mean of precision and recall, offering a single metric that considers both aspects of performance; the area under the receiver operating characteristic (roc) curve, which demonstrates the degree or measure of separability of the classes, and the mean normalized confusion matrix, which provides us the distribution of error within the classes.

ML Model Explainability. To interpret the model’s output and understand the contribution of each feature, we employed the SHapley Additive exPlanations (SHAP) model. SHAP values provide a unified measure of feature importance by attributing the prediction made by the model to individual features. This model is based on Game Theory and enhances our understanding of the intricate relationships within the data, revealing how different features influence the likelihood of outcomes.

All code for ML model development, training, and evaluation was written in Python, with the Scikit-learn library (https://scikit-learn.org/, accessed on 30 July 2024) serving as the primary tool for implementing ML algorithms and techniques.

### 2.6. Statistical Analysis

The statistical analysis was conducted using the IBM SPSS statistical package, version 29. Descriptive statistics were calculated to summarize the data. The Kolmogorov–Smirnov test was applied to assess the normality of the chronological age, BMI and age at PHV based on Mirwald and the employed selected variables based on the SFFS FS algorithm. All the employed variables present normal distributions. To compare the differences in the continuous predictors between groups ‘predicted age at PHV ≤ median’ and ‘predicted age at PHV > median’, an independent sample *t*-test was utilized. In order to estimate the effect size, Cohen’s d was also calculated.

## 3. Results

### 3.1. Descriptive Statistics

Table 4 presents the descriptive statistics of the subjects, categorized based on whether their predicted age at peak height velocity (PHV) is below or above the median (−1.7665). This median value represents the midpoint of the sample, dividing the participants into two groups for comparison purposes. For subjects with predicted age at PHV ≤ median (n = 47), the mean chronological age was 11.68 years (SD = 0.29), and the mean body mass index (BMI) was 18.30 (SD = 2.66). The mean age at PHV Mirwald value for this group was −2.24 (SD = 0.34). In contrast, subjects with predicting age at PHV > median (n = 46) had a mean chronological age of 11.89 years (SD = 0.25), a mean BMI of 20.68 (SD = 3.37), and a mean age at PHV Mirwald value of −1.26 (SD = 0.41).

### 3.2. Testing Performance

Table 5 presents a summary of the testing performance metrics in this binary task, including the averaged accuracy, recall, precision, F1-score and area under the ROC curve for the employed ML classifiers. The aforementioned metrics were derived by individually applying the employed FS algorithm to each ML classifier. The LR classifier demonstrated the best performance compared to the others, achieving an accuracy of 96.67%, a recall of 98.00%, a precision of 96.33%, an F1-score of 97.09% and an area under the ROC curve of 99.00%. On the other hand, the RF classifier exhibited the lowest performance metrics, with an accuracy of 94.44% and an area under the ROC curve of 96.25%.

**Figure 1:**
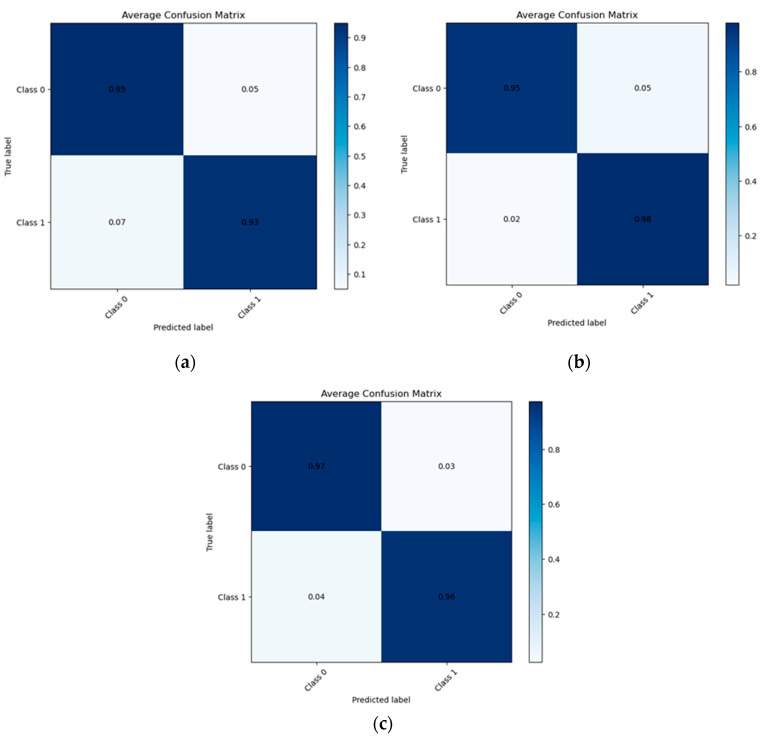
Confusion matrixes for the employed ML classifiers: (**a**) RF, (**b**) LR and (**c**) NN classifier.

Figure 1 depicts the normalized confusion matrixes of the employed classifiers. Specifically, the LR classifier achieved 0.98 sensitivity and 0.95 specificity in this binary task.

### 3.3. Feature Selection

Table 6 displays the most informative factors that were identified for the binary classification task of distinguishing children with predicted age at PHV ≤ median from children with predicting age at PHV > median for the top-performing LR classifier.

### 3.4. Interpretation

**Figure 2:**
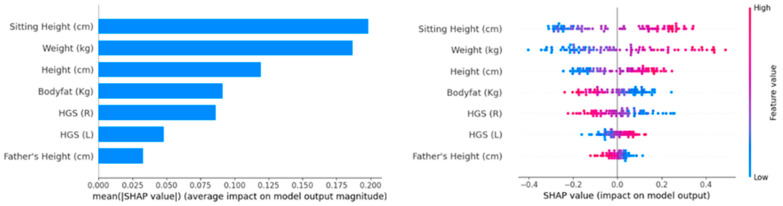
SHAP analysis for binary classification task using LR model. This figure presents the LR classifier model’s output, (**a**) the mean SHAP values for feature importance and (**b**) the SHAP summary plot for feature impact.

To interpret the model output of the best-performing ML classifier (LR model) and understand the contribution of each factor, the SHAP model was used. SHAP values provide a unified measure of feature importance by attributing the prediction made by the model to individual features. Figure 2a,b illustrate the SHAP analysis results. Figure 2a shows the mean SHAP value for each feature, which represents the average impact on the model output magnitude for both classes (PHV ≤ median and PHV > median class). This figure highlights that ‘Sitting Height (cm)’ and ‘Weight (kg)’ are the most influential features, followed by ‘Height (cm)’, ‘Body Fat (kg)’, ‘HGS (R)’, ‘HGS (L)’, and ‘Father’s Height (cm)’. These features exhibit the highest SHAP values, indicating their significant contribution to the model’s predictions. 

Figure 2b provides a detailed scatter plot of SHAP values for individual predictions. Each dot represents a sample, and the color indicates the feature value (with red representing high values and blue representing low values). The horizontal axis shows the SHAP value, depicting the impact on the model output. This figure further emphasizes the importance of ‘Sitting Height (cm)’ and ‘Weight (kg)’ as the features with the most substantial impact on the model output across different samples. Additionally, it shows the distribution of SHAP values, indicating how changes in feature values influence the predictions. For ‘Sitting Height (cm)’, higher values (indicated by red dots) push the model towards predicting PHV > median class, as evidenced by positive SHAP values. This suggests that individuals with greater sitting height are more likely to be classified into PHV > median class. Similarly, ‘Weight (kg)’ and ‘Height (cm)’ follow the same pattern, with higher weights and heights (also shown in red) driving the predictions towards PHV > median class. The SHAP values for ‘Weight (kg)’ and ‘Height’ show a positive correlation with the model output for the PHV > median class, meaning that as weight and height increase, the likelihood of being classified into the PHV > median class also increases. ‘Body Fat (kg)’ also contributes significantly to the model’s prediction. Higher ‘Body Fat (kg)’ (red dots) results in negative SHAP values, indicating a higher probability of being classified as PHV ≤ median class. The feature ‘HGS (L)’, while still impactful, shows a less pronounced but similar trend, where greater heights push the model towards PHV > median class, and the feature ‘HGS (R)’ shows a less pronounced but similar trend where greater heights push the model towards PHV ≤ median class. Finally, the feature ‘Father’s Height (cm)’ presents a neutral contribution.

To summarize, the positive SHAP values for these features (Sitting Height (cm), Weight (kg), Height (cm) and Body Fat (kg)) indicate their strong influence in favoring PHV > median class predictions. The clustering of red dots (high feature values) on the positive side of the SHAP value axis underscores how higher measurements in these features consistently lead to a higher likelihood of the model predicting PHV > median class.

### 3.5. Statistical Analysis

Table 7 depicts the results of the independent *t*-test statistical analysis of the most informative factors selected. Specifically, the results indicate significant differences between participants with predicted age at peak height velocity (PHV) less than or equal to the median and those with predicted age at PHV greater than the median in several anthropometric and physical characteristics. Specifically, participants with predicted age at PHV > median exhibited higher sitting height (81.87 ± 3.07 cm vs. 74.98 ± 2.97 cm, *p* < 0.001, d = 3.02), father’s height (181.08 ± 6.36 cm vs. 178.05 ± 5.72 cm, *p* < 0.05, d = 6.05), height (160.68 ± 5.71 cm vs. 148.67 ± 5.88 cm, *p* < 0.001, d = 5.79), body fat (13.47 ± 6.27 kg vs. 9.50 ± 4.86 kg, *p* < 0.001, d = 5.60), and weight (53.44 ± 9.47 kg vs. 40.45 ± 6.89 kg, *p* < 0.001, d = 8.27). Additionally, handgrip strength was significantly greater in the predicted age at PHV > median group for both the left hand (22.11 ± 4.14 N vs. 18.78 ± 3.45 N, *p* < 0.001, d = 3.81) and the right hand (23.04 ± 3.98 N vs. 19.57 ± 3.57 N, *p* < 0.001, d = 3.78).

## 4. Discussion

This work aims to develop a methodology that leverages XAI techniques to identify the key factors influencing peak height velocity in pre-adolescent team sports athletes. We employed three well-known classifiers and applied an SFFS algorithm to each. In this binary classification task, the LR classifier achieved the highest accuracy of 96.67% with the seven most informative factors. The SHAP method was crucial in determining the significance of each factor in the model’s output, providing valuable insights into the contributions of different variables. To validate our findings, we conducted independent *t*-tests on the selected informative factors, confirming their importance in distinguishing between the two classes. This comprehensive approach underscores the potential use of explainable machine learning techniques in uncovering critical determinants for the prediction of age at peak height velocity.

Initially, the SFFS FS technique identified sitting height, weight, height, body fat, handgrip strength (both left and right), and father’s height as the most informative factors for predicting age at peak height velocity (PHV). These selected factors align well with the existing literature on growth and maturation during adolescence. Malina et al. [34] highlighted that anthropometric characteristics like height and weight are significantly influenced by age and maturation, particularly during the rapid growth phases in adolescence. Kalcikova and Pridalova [35] further support the importance of body composition indicators, noting the marked differences in body fat mass pre- and post-PHV. Handgrip strength, as noted by Loyd et al. [36], becomes a crucial performance indicator during puberty due to both neural and muscular changes. Father’s height, a predictor of future body size, is also corroborated by Lolli et al. [37] as a useful metric in athletic populations. Thus, the selection of these factors by the FS technique is well-supported by the literature, emphasizing their relevance in estimating PHV.

SHAP analysis provided insights into how each feature contributed to the model’s predictions. Sitting height and weight were the most influential, with higher values strongly pushing the model towards predicting PHV > median class. This observation is consistent with those in the literature, stating that during the PHV period, there is a significant increase in height and weight [34]. The SHAP values for body fat showed a negative correlation with PHV > median class predictions, aligning with findings by Kalcikova and Pridalova [35] that body fat percentage decreases during this growth phase. The less pronounced but still significant contributions of handgrip strength also fit well with the findings of Loyd et al. [36], who noted the non-linear strength gains during puberty. Father’s height’s neutral contribution, although included, may reflect the complex interplay of genetic and environmental factors in growth. Overall, the SHAP interpretation confirms the literature’s findings, highlighting the critical role of these features in growth and maturation during adolescence.

Furthermore, the statistical analysis results reinforce the significance of the selected factors in distinguishing between different PHV groups. Participants with predicted age PHV > median had significantly higher sitting height, weight, height, body fat, and handgrip strength. These differences, supported by *p*-values and effect sizes, mirror the documented trends in the literature. The significant differences in sitting height and weight are in line with the findings of Malina et al. [34], who described substantial growth in these parameters during adolescence. The higher body fat in the predicted age at PHV > median group, despite a general trend of decreasing body fat percentage, may indicate varying individual growth patterns, as noted by Kalcikova and Pridalova [35]. The increased handgrip strength in the predicted age at PHV > median group aligns with the accelerated strength gains during puberty discussed by Loyd et al. [36]. Lastly, the taller father’s height in the predicted age at PHV > median group supports the genetic influence on growth, as noted by Lolli et al. [37]. Thus, the statistical results corroborate those in the literature, validating the AI-identified factors’ relevance and importance in predicting age at PHV.

In addition, genetic factors are well-established as the most significant contributors to final height, but the secular trend of growth is strongly influenced by environmental factors [38]. There is a strong correlation between access to healthcare, nutritional status, and stature [39]. Additionally, education, income, and social status are influential factors that interact throughout the growth period, contributing to population growth trends [40,41]. In the same vein, Arntsen et al. provided evidence that when poverty decreased and healthcare and nutrition improved, overall growth and adult height increased. A remarkable finding was that mean body height increased by 3.4 cm over 47 years (for individuals born between 1930 and 1977) [42], while an even larger increase of 10.8 cm was observed over a period of 122 years. This demonstrates that environmental factors can significantly influence stature over time, alongside genetics. In populations where socioeconomic conditions, such as in the United States, improved substantially, notable secular increases in height were observed. In contrast, only small increases in stature were recorded in Europe between 1760 and 1830, likely due to modest improvements in nutrition and healthcare during that period [43]. As mentioned earlier, maturation can vary between peers and different body parts [12]. Our findings align with those of previous research, confirming that trunk growth occurs later than growth in lower body extremities [44], making sitting height an important tool for identifying individual differences. Heritability has been established as one of the most influential factors for variables like stature [45]. In agreement with the results of Su et al., this study also demonstrates that a father’s height significantly contributes to the son’s height, particularly in the case of taller sons [46]. In conclusion, the key parameters identified using AI models are supported by various studies, confirming their integral role in accurately predicting maturity.

Despite the equations for predicting age at PHV and predicted adult height being well-documented and widely used, individual variability can impact the accuracy of these predictions. Therefore, incorporating additional methods could provide a more robust estimation of the peak height velocity period. A potential limitation is the reliance on participants’ honesty regarding their training sessions. Any discrepancies could affect body weight and composition measurements, as inactivity is directly correlated with increases in body weight and body fat. This reliance on self-reported data introduces an element of uncertainty that must be considered when interpreting the results. Furthermore, genes are considered one of the most important factors influencing growth and maturation; however, the environment in which a child is born and raised plays a significant role in determining whether they will reach their potential growth [9]. These factors need to be assessed longitudinally, making it difficult to control for variability between subjects. Dietary behavior is another potential limitation in studies that include body composition parameters [47]. A related factor is hydration, which is critical for daily performance, as approximately 70% of the human body is composed of fluids and hydration levels can influence body composition values [48]. Research has shown an association between hydration, weight status, and BMI in children and adolescents [49], making hydration a potential limitation in self-reported data.

In addition, our power analysis indicated that a similar number of subjects was necessary to ensure the study’s statistical validity and the robustness of its findings.

To summarize, this study will help coaches and sports specialists to identify talented players more accurately and not overestimate or underestimate an athlete’s current performance. Specifically, athletes that are biologically older experience accelerations in stature development and physical fitness characteristics earlier in their life [50]. Moore et al. have mentioned that early-matured males can perform better in sports like soccer, since they are bigger in body size and produce more muscle strength power [51]. In that case, chronological age can be a misleading factor. In addition, the magnitude of maturation differences between individuals of the same chronological group tends to be greater at the national selection level than at the club level [52]. As a result, the findings of the study can be used in the future for talent selection to avoid disadvantages to late-maturing children and adolescents, who have a small chance of reaching the elite level, although their potential peak performance may exceed that of early matured athletes [53].

For future research, the reassessment of the participants at one year or even later to validate the predictions and measure the actual age at peak height velocity would be of importance. This follow-up will allow the comparison of the predicted values with real-world data, enhancing the accuracy and reliability of the current findings. Continuously monitoring the participants’ growth patterns and adjusting the predictive models accordingly would help refine our understanding of PHV and its determinants. This longitudinal approach will also help identify any long-term effects of training exposure on growth and body composition, providing more comprehensive insights into adolescent development. By focusing on longitudinal data collection, future work will contribute to more accurate and individualized growth predictions, ultimately benefiting both health and sports performance outcomes. In addition to the longitudinal approach, future research could focus on expanding the sample to include athletes from other sports, such as swimming, tennis, and handball, which are also popular among children. Collecting data from a broader geographical area would strengthen the applicability of the findings and provide a more representative view of the characteristics of 11-year-old athletes in Greece. It is also important to consider female populations in future studies, allowing for comparisons between boys and girls.

## 5. Conclusions

The findings of this study underscore the robustness and reliability of the methodology used in the present study, which utilizes XAI techniques to identify critical determinants of PHV in children aged 11. By focusing on the key factors that differentiate children with predicted age at PHV below and above the median, this approach highlights the potential of combining advanced XAI models with traditional growth assessment methods. This integration offers precise and individualized insights into child development, paving the way for more effective interventions in physical performance, health monitoring and growth management. Specifically, predicting PHV can be useful for preventing injuries and improving talent selection. During adolescence, limbs grow at a faster rate, which can lead to delays and regressions in motor control, increasing the risk of injury. Supporting this idea, research has shown that injured adolescent athletes often experience rapid growth compared to their non-injured counterparts. On the other hand, talent identification is considered crucial for promoting long-term athletic development. However, more physically advanced youth athletes within the same chronological age group are often incorrectly identified as more talented, which reflects a temporary advantage in performance but does not necessarily indicate long-term superior athletic potential. Using this information, youth strength and conditioning trainers and coaches will be better equipped to prescribe the appropriate ‘dose’ of physical activity, adjusting factors such as frequency, intensity, and duration of exercise, whether during on-court sessions or in the weight room, to maximize maturation outcomes. For instance, during periods of accelerated growth, it is more effective to focus on technique and quality of movement rather than the quantity or volume of training. Rapid physical changes also affect the training load an individual can tolerate. Professionals should progress gradually, increasing training frequency or maintaining/decreasing exposure when appropriate. Furthermore, youth strength and conditioning trainers and coaches can make more informed decisions about when to introduce specific training elements. These advancements hold promise for revolutionizing pediatric growth analysis, ultimately contributing to improved outcomes for children’s health and development.

## Figures and Tables

**Table 1:** Anthropometric characteristics.

Variable	Mean Value	Standard Deviation
Chronological Age (y)	11.71	0.34
Height (cm)	154.61	8.34
Father’s Height (cm)	179.55	6.34
Mother’s Height (cm)	167	5.16
Sitting Height (cm)	78.38	4.58
Leg Length (cm)	76.22	4.43
Weight (kg)	46.87	10.5
Age at Peak Height Velocity (Mirwald)	−1.75	0.61

**Table 2:** Body composition.

Variable	Mean Value	Standard Deviation
Body Mass Index	19.47	3.24
Body Fat (%)	23.33	8.47
Body Fat (kg)	11.46	5.91
Lean Mass (kg)	33.6	8.09

**Table 3:** Performance tests.

Variable	Mean Value	Standard Deviation
Handgrip Strength—Right Hand (n)	21.2	4.1
Handgrip Strength—Left Hand (n)	20.4	4.1
Countermovement Jump (cm)	21.2	4.5
Countermovement Jump—With Arm Swing (cm)	24.8	4.6

**Table 4:** Descriptive statistics of the employed subjects (mean ± SD).

	Predicted Age at PHV ≤ Median (n = 47)	Predicted Age at PHV > Median (n = 46)	Sig. (*p*-Value)	Cohen’s d
Chronological Age	11.68 ± 0.29	11.71 ± 0.34	0.17	0.34
BMI	18.30 ± 2.66	20.68 ± 3.37	<0.001	3.03
PHV Based on Mirwald	−2.24 ± 0.34	−1.26 ± 0.41	<0.001	0.37

**Table 5:** Testing performance of the employed ML classifiers.

Classifiers	Accuracy (%)	Recall (%)	Precision (%)	F1-Score (%)	ROC AUC (%)	Num. of Selected Features
RF	94.44	93.50	95.83	94.33	96.25	2
LR	96.67	98.00	96.33	97.09	99.00	7
NN	96.67	95.50	98.33	96.55	98.00	5

**Table 6:** The most informative factors are based on the SFFS FS technique for the LR classifier.

Feature	Type of Variable
Sitting Height (cm)	Numeric
Father’s Height (cm)	Numeric
Body Fat (kg)	Numeric
Weight (kg)	Numeric
Height (cm)	Numeric
HGS (L)	Numeric
HGS (R)	Numeric

**Table 7:** Results of the statistical analysis.

	Predicted Age at PHV ≤ Median (Mean ± SD)	Predicted Age at PHV > Median (Mean ± SD)	Sig. (*p*-Value)	Cohen’s d
Sitting Height (cm)	74.98 ± 2.97	81.87 ± 3.07	<0.001	3.02
Father’s Height (cm)	178.05 ± 5.72	181.08 ± 6.36	<0.05	6.05
Height (cm)	148.67 ± 5.88	160.68 ± 5.71	<0.001	5.79
Body Fat (kg)	9.50 ± 4.86	13.47 ± 6.27	<0.001	5.60
Weight (kg)	40.45 ± 6.89	53.44 ± 9.47	<0.001	8.27
HGS_L (N)	18.78 ± 3.45	22.11 ± 4.14	<0.001	3.81
HGS_R (N)	19.57 ± 3.57	23.04 ± 3.98	<0.001	3.78

Significant differences (*p*-value) < 0.05.

## Data Availability

The data used in this study, which include information about minors, are confidential and cannot be shared due to stringent privacy regulations and ethical considerations. Access to the data is strictly restricted to the research team so as to protect the identity and well-being of the participants.

## References

[1] Moore S.A. (2018). Assessing Somatic Maturity in Children and Adolescents: Relevance to Pediatric Bone Health. Ph.D. Thesis.

[2] Romann M. (2020). Improving Talent Identification Through Analysis and Consideration of Biological and Relative Age. https://www.researchgate.net/publication/347240903_Improving_talent_identification_through_analysis_and_consideration_of_biological_and_relative_age.

[3] Kezic A., Babic M., Cular D. (2024). Maturity Status and Relative Age of Elite Taekwondo Youth Competitors—Case Study on Croatian National Team. Sports.

[4] Sellami M., Makni E., Moalla W., Tarwneh R., Elloumi M. (2024). Effect of Maturation Level on Normative Specific-Agility Performance Metrics and Their Fitness Predictors in Soccer Players Aged 11–18 Years. BMC Sports Sci. Med. Rehabil..

[5] Enrique Carranza-García L., Cervantes-Hernández N., Domínguez-Sosa M., Alanís-Flores M., López-García R., Vasquez-Bonilla A., Alberto Flores L. (2024). Somatic Maturity and Physical Performance in Male Youth Players from a Professional Soccer Academy. Int. J. Morphol..

[6] Malina R.M., Kozieł S.M., Králik M., Chrzanowska M., Suder A. (2021). Prediction of Maturity Offset and Age at Peak Height Velocity in a Longitudinal Series of Boys and Girls. Am. J. Hum. Biol..

[7] Bult H.J., Barendrecht M., Tak I.J.R. (2018). Injury Risk and Injury Burden Are Related to Age Group and Peak Height Velocity Among Talented Male Youth Soccer Players. Orthop. J. Sports Med..

[8] Toselli S., Grigoletto A., Zaccagni L., Rinaldo N., Badicu G., Grosz W.R., Campa F. (2021). Body Image Perception and Body Composition in Early Adolescents: A Longitudinal Study of an Italian Cohort. BMC Public Health.

[9] Armstrong N., Van Mechelen W. (2017). Oxford Textbook of Children’s Sport and Exercise Medicine.

[10] Malina R.M., Rogol A.D., Cumming S.P., e Silva M.J.C., Figueiredo A.J. (2015). Biological Maturation of Youth Athletes: Assessment and Implications. Br. J. Sports Med..

[11] Malina R.M. (2014). Top 10 Research Questions Related to Growth and Maturation of Relevance to Physical Activity, Performance, and Fitness. Res. Q. Exerc. Sport.

[12] Faigenbaum A.D., Lloyd R.S., Oliver J.L. (2019). Essentials of Youth Fitness.

[13] Caine D., Purcell L., Maffulli N. (2014). The Child and Adolescent Athlete: A Review of Three Potentially Serious Injuries. BMC Sports Sci. Med. Rehabil..

[14] Roemmich J.N., Richmond E., Rogol A. (2001). Consequences of Sport Training during Puberty. J. Endocrinol. Investig..

[15] Nobari H., Silva A.F., Clemente F.M., Siahkouhian M., García-Gordillo M.Á., Adsuar J.C., Pérez-Gómez J. (2021). Analysis of Fitness Status Variations of Under-16 Soccer Players over a Season and Their Relationships with Maturational Status and Training Load. Front. Physiol..

[16] Hussey C., Gupta A. (2023). Importance of Physical Activity and Its Impact on Growth, Development and Health. Young People Practising Health Educational Tools for Wellbeing Through Sport and Nutrition.

[17] Dudley C., Johnston R., Jones B., Till K., Westbrook H., Weakley J. (2023). Methods of Monitoring Internal and External Loads and Their Relationships with Physical Qualities, Injury, or Illness in Adolescent Athletes: A Systematic Review and Best-Evidence Synthesis. Sports Med..

[18] Mirwald R.L., Baxter-Jones A.D., Bailey D.A., Beunen G.P. (2002). An Assessment of Maturity from Anthropometric Measurements. Med. Sci. Sports Exerc..

[19] Moore S.A., McKay H.A., Macdonald H., Nettlefold L., Baxter-Jones A.D., Cameron N., Brasher P.M. (2015). Enhancing a Somatic Maturity Prediction Model. Med. Sci. Sports Exerc..

[20] Tsutsui T., Iizuka S., Sakamaki W., Maemichi T., Torii S. (2022). Growth until Peak Height Velocity Occurs Rapidly in Early Maturing Adolescent Boys. Children.

[21] Malina R.M., Coelho-e-Silva M.J., Martinho D.V., Sousa-e-Siva P., Figueiredo A.J., Cumming S.P., Králík M., Kozieł S.M. (2021). Observed and Predicted Ages at Peak Height Velocity in Soccer Players. PLoS ONE.

[22] Kleanthous K., Papadimitriou D.T., Gryparis A., Papaevangelou V., Papadimitriou A. (2022). A Mixed-Longitudinal Study of Height Velocity of Greek Schoolchildren and the Milestones of the Adolescent Growth Spurt. Children.

[23] Shmoish M., German A., Devir N., Hecht A., Butler G., Niklasson A., Albertsson-Wikland K., Hochberg Z. (2021). Prediction of Adult Height by Machine Learning Technique. J. Clin. Endocrinol. Metab..

[24] Zhang L., Chen J., Hou L., Xu Y., Liu Z., Huang S., Ou H., Meng Z., Liang L. (2022). Clinical Application of Artificial Intelligence in Longitudinal Image Analysis of Bone Age among GHD Patients. Front. Pediatr..

[25] Plakias S., Kokkotis C., Mitrotasios M., Armatas V., Tsatalas T., Giakas G. (2024). Identifying Key Factors for Securing a Champions League Position in French Ligue 1 Using Explainable Machine Learning Techniques. Appl. Sci..

[26] Plakias S., Kokkotis C., Pantazis D., Tsatalas T. (2024). Comparative Analysis of Key Performance Indicators in Euroleague and National Basketball Leagues. J. Phys. Educ. Sport.

[27] Moustakidis S., Plakias S., Kokkotis C., Tsatalas T., Tsaopoulos D. (2023). Predicting Football Team Performance with Explainable Ai: Leveraging Shap to Identify Key Team-Level Performance Metrics. Future Internet.

[28] Kokkotis C., Moustakidis S., Tsatalas T., Ntakolia C., Chalatsis G., Konstadakos S., Hantes M.E., Giakas G., Tsaopoulos D. (2022). Leveraging Explainable Machine Learning to Identify Gait Biomechanical Parameters Associated with Anterior Cruciate Ligament Injury. Sci. Rep..

[29] Weiner J.S., Lourie J.A. (1969). Human Biology, A Guide to Field Methods.

[30] Gerodimos V. (2012). Reliability of Handgrip Strength Test in Basketball Players. J. Hum. Kinet..

[31] Barazetti L.K., Varoni P.R., Campos F.d.S., Demarchi M., Baumann L., Teixeira A.S., Nunes R.F.H., Flores L.J.F. (2019). Comparison of Maturation and Physical Performance in Basketball Athletes of Different Playing Positions. Rev. Bras. Cineantropometria Desempenho Hum..

[32] Cabarkapa D., Philipp N., Cabarkapa D., Eserhaut D., Fry A. (2023). Comparison of Force-Time Metrics between Countermovement Vertical Jump with and without an Arm Swing in Professional Male Basketball Players. Int. J. Strength Cond..

[33] Sebastian R., Vahid M. (2019). Python Machine Learning: Machine Learning and Deep Learning with Python, Scikit-Learn, and TensorFlow 2.

[34] Malina R.M., Eisenmann J.C., Cumming S.P., Ribeiro B., Aroso J. (2004). Maturity-Associated Variation in the Growth and Functional Capacities of Youth Football (Soccer) Players 13–15 Years. Eur. J. Appl. Physiol..

[35] Kalčíková P., Přidalová M. (2023). The Influence of Somatic Maturity on Anthropometrics and Body Composition in Youth Soccer Players. Children.

[36] Lloyd R.S., Faigenbaum A.D., Stone M.H., Oliver J.L., Jeffreys I., Moody J.A., Brewer C., Pierce K.C., McCambridge T.M., Howard R. (2014). Position Statement on Youth Resistance Training: The 2014 International Consensus. Br. J. Sports Med..

[37] Lolli L., Johnson A., Monaco M., Cardinale M., Di Salvo V., Gregson W. (2021). Tanner–Whitehouse and Modified Bayley–Pinneau Adult Height Predictions in Elite Youth Soccer Players from the Middle East. Med. Sci. Sports Exerc..

[38] Fudvoye J., Parent A.-S. (2017). Secular Trends in Growth.

[39] Tanner J.M. (1992). Growth as a Measure of the Nutritional and Hygienic Status of a Population. Horm. Res. Paediatr..

[40] Meyer H.E., Selmer R. (1999). Income, Educational Level and Body Height. Ann. Hum. Biol..

[41] Kuh D.L., Power C., Rodgers B. (1991). Secular Trends in Social Class and Sex Differences in Adult Height. Int. J. Epidemiol..

[42] Arntsen S.H., Borch K.B., Wilsgaard T., Njølstad I., Hansen A.H. (2023). Time Trends in Body Height According to Educational Level. A Descriptive Study from the Tromsø Study 1979–2016. PLoS ONE.

[43] Roche A.F. (1979). Secular Trends in Stature, Weight, and Maturation. Monogr. Soc. Res. Child Dev..

[44] Baxter-Jones A.D., Thompson A.M., Malina R.M. (2002). Growth and Maturation in Elite Young Female Athletes. Sports Med. Arthrosc. Rev..

[45] Brener A., Waksman Y., Rosenfeld T., Levy S., Peleg I., Raviv A., Interator H., Lebenthal Y. (2021). The Heritability of Body Composition. BMC Pediatr..

[46] Su P.-H., Wang S.-L., Chen J.-Y. (2011). Gender Differences of Final Height Contributed by Parents’ Height among Healthy Individuals. Pediatr. Neonatol..

[47] Kobylińska M., Antosik K., Decyk A., Kurowska K., Skiba D. (2022). Body Composition and Anthropometric Indicators in Children and Adolescents 6–15 Years Old. Int. J. Environ. Res. Public. Health.

[48] Ashadi K., Fachri R.L., Siantoro G., Kusuma D.A., Hariyanto A., Kusuma I.D.M. (2018). Comparison of Knowledge and Hydration Awareness on Adolescent Soccer Athletes.

[49] Clayton P., Trak-Fellermeier M.A., Macchi A., Galván R., Bursac Z., Huffman-Ercanli F., Liuzzi J., Palacios C. (2023). The Association between Hydration Status and Body Composition in Healthy Children and Adolescents. J. Pediatr. Endocrinol. Metab..

[50] MacMaster C. Maturity-Status ‘Bio-Banding’ as a Tool for Ongoing Talent (de) Selection of Academy Soccer Players Using a Multi-Disciplinary Approach. https://hull-repository.worktribe.com/output/4222839/maturity-status-bio-banding-as-a-tool-for-ongoing-talent-deselection-of-academy-soccer-players-using-a-multi-disciplinary-approach.

[51] Moore S., Moore M., Klentrou P., Sullivan P., Falk B. (2010). Maturity Status in Male Child and Adolescent Athletes. J. Sports Med. Phys. Fitness.

[52] Sweeney L., Cumming S.P., MacNamara Á., Horan D. (2023). A Tale of Two Selection Biases: The Independent Effects of Relative Age and Biological Maturity on Player Selection in the Football Association of Ireland’s National Talent Pathway. Int. J. Sports Sci. Coach..

[53] Müller L., Müller E., Hildebrandt C., Kapelari K., Raschner C. (2015). The Assessment of Biological Maturation for Talent Selection-Which Method Can Be Used?. Sportverletz. Sportschaden Organ Ges. Orthopadisch-Traumatol. Sportmed..

